# The role of expanded close contact screening in the tuberculosis outbreak at a school in China

**DOI:** 10.3389/fpubh.2025.1655711

**Published:** 2025-09-04

**Authors:** Liai Peng, Jinzhou Mei, Fangxiang Hu, Mingbin Xie, Zhenyang Liu, Yanfang Guo, Chongguang Yang, Yunxia Wang

**Affiliations:** ^1^Department of Tuberculosis Control and Prevention, Bao'an District Hospital for Chronic Diseases Prevention and Cure, Shenzhen, Guangdong, China; ^2^School of Public Health (Shenzhen), Shenzhen Campus of Sun Yat-sen University, Shenzhen, Guangdong, China

**Keywords:** tuberculosis, school, cluster outbreak, close contact screening, public health management

## Abstract

**Background:**

Tuberculosis (TB) outbreaks in confined settings such as schools pose significant public health challenges due to the potential for rapid transmission among closely interacting individuals. In December 2018, a senior high school student in Shenzhen City, China, was diagnosed with etiological positive TB, prompting an investigation that extended until November 2024. This study aimed to analyze the outbreak’s characteristics, identify its causes, and provide insights for timely identification and management of similar clusters.

**Methods:**

The confirmed, clinically diagnosed, and suspected cases of TB were identified according to the “Tuberculosis diagnosis WS288-2017” criteria. Epidemiological investigations of TB cases included close contact screening via symptom assessment, TST, and chest radiography. Moderately TST-positive contacts underwent IGRA confirmation for preventive therapy eligibility, while MIRU-VNTR genotyping of culture-positive isolates delineated transmission networks. The Chi-square test or Fisher’s exact test was employed to analyze changes in TST positivity rates and differences in TB incidence rates.

**Results:**

A total of six TB cases were detected in the high school, with five screenings conducted over the study period. Misdiagnosis caused a near-three-month delay from symptom onset to confirmed TB in the index case. Among the five newly diagnosed patients, four were in the same class as the index case, and one was in an adjacent class. These two classes are located on the middle horizontal line of the “B”-shaped teaching building. For the indicated case’s class, the positive rate of TST in the second screening (35.85, 95% CI: 23.49–19.25%) was significantly higher than in the first screening (8.93, 95% CI: 3.33–20.37%) (χ^2^ = 11.493, *p* < 0.001). MIRU-VNTR genotyping of four clinical isolates identified concordant non-Beijing strains with matching profiles at 11/12 loci (excluding VNTR3232), demonstrating a single transmission chain.

**Conclusion:**

This outbreak was a cluster epidemic driven by misdiagnosis, poor ventilation, and insufficient routine prevention measures. Establishing long-term close-contact monitoring and secondary screening is crucial for identifying infections missed during the initial window period, thereby mitigating the spread of TB in similar settings and improving outbreak management strategies.

## 1 Background

Tuberculosis (TB) remains a formidable global health challenge, ranking among the top 10 causes of death worldwide (1). According to the 2024 Global TB Report, in 2023, approximately 10.8 million new TB cases were reported, with an estimated 1.25 million deaths attributed to TB. With 6.8% of the global TB cases, China is positioned third among the high TB burden countries (1). Previous studies have indicated that TB outbreaks are more likely to occur in specific populations and settings, such as refugees (2), immigrants (3, 4), prisons (5), internet cafes (6), offices (7), and schools (8, 9).

School settings present unique epidemiological risks due to high population density, prolonged close contact, and adolescents’ immunological vulnerability (9, 10). In China, the student population is considerable, comprising nearly one-fifth of the total population. Over the period from 2004 to 2021, 908,171 TB cases were reported among students, accounting for 5.37% of the total number of reported TB cases in the general population (16,902,179 cases) (11). Notably, the proportion of reported TB cases among students has been on a steady upward trend (12). Between 2015 and 2019, the reported incidence rate of TB among students increased by nearly 30% (11, 13). Research findings indicate that high schools are the most common sites of TB outbreaks (14, 15). Data from the China Disease Prevention and Control Information System reveal that from 2017 to 2019, 48 TB outbreaks were reported in schools, of which 36 occurred in high schools (15). Compared to other school settings, boarding schools are subject to a higher risk of TB outbreaks, where adolescents often cluster under relatively overcrowded conditions (14, 16). The frequent occurrence of outbreaks, especially within schools, is one of the essential reasons for the high incidence of TB in China (17). This issue is not unique to China, and other countries around the world also face similar challenges with school TB outbreaks (18–20).

On December 11, 2018, a student in a high school in Shenzhen City, Guangdong province, China, was diagnosed as a TB case with positive sputum culture. We conducted a series of screenings and investigations into the outbreak at the school and have been monitoring it up to November 2024. Throughout the subsequent observation, five additional cases of TB were identified among the students in the patient’s class and the neighboring class. We retrospectively analyzed the survey data to analyze the characteristics and potential causes of this TB cluster outbreak and provide insights for timely identification and management.

## 2 Methods

### 2.1 Study setting and study design

**Figure 1:**
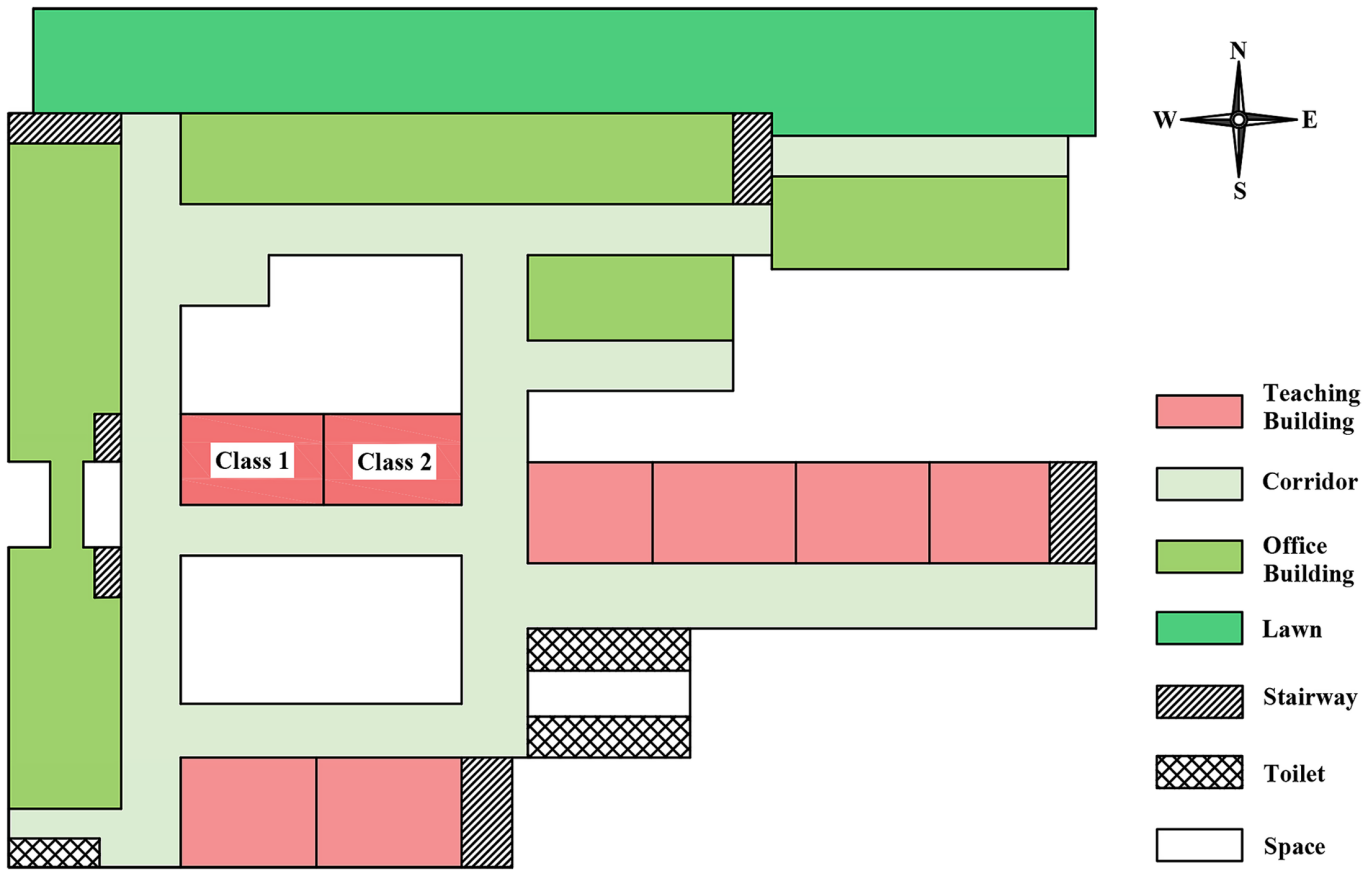
Senior high school teaching area layout.

The outbreak occurred at a public high school that accommodates boarding students. It is segregated into junior and senior high school sections, which are located at considerable distances from each other. The school had 3,710 total students, including 1,860 senior high students distributed across 36 classes (average 52 students / class). The teaching buildings at the senior high school were configured in a “B” shape (Figure 1). The school’s boarding program was exclusively for senior high students, with a total of 1,435 boarding students accommodated. Two student dormitory buildings were present, with the lower floors (first to fourth) designated for boys and the upper floors (fifth to seventh) for girls. Each dormitory housed eight students.

The outbreak was confined to the senior high school division. We retrospectively reviewed the outbreak survey data, investigation records, and medical records. This included detailed information on the students and school, such as gender, classroom and residential status, classroom and dormitory distributions, tuberculin skin test (TST) results, and clinical information of TB cases. Additionally, we interviewed physicians and nurses from the school health clinic. We also conducted in-person interviews of case-students or telephoned their parents to gather fundamental case history, time of disease onset, how medical services were sought, diagnosis, and treatment.

### 2.2 Diagnosis and definitions

All TB cases, including laboratory-confirmed cases, clinically diagnosed cases, and suspected cases, were defined and classified following the Diagnostic Criteria for Pulmonary TB (WS 288–2017) and Classification of TB (WS 196-2017) in China (21, 22). Among these, suspected cases were defined as individuals presenting radiological features consistent with TB, representing pre-diagnostic cases requiring further evaluation.

The initial case of active TB identified within the school was defined as the index case. Subsequently, contacts were categorized into three groups—close contacts, general contacts, and occasional contacts—based on the mode, degree, and duration of their exposure to the index case. Among them, close contacts referred to individuals who had had direct contact with TB patients, including teachers and students sharing the same classroom and the same dormitory (23).

For TST, an induration with a mean diameter <5 mm was classified as negative; 5–<10 mm as generally Mantoux positive; 10–<15 mm as moderately Mantoux positive. A strong Mantoux positive (SMP) reaction was defined as an induration ≥15 mm and/or with blisters, necrosis, and lymphangitis. A positive TST result was described as an induration ≥5 mm.

### 2.3 Contacts screening

Five contact screenings were performed according to the school TB prevention and control work regulations (2017 edition) (23), which included symptom screening, TST, and chest radiography. Individuals under the age of 15 underwent symptom screening and TST initially. Subsequently, chest radiography was conducted on those exhibiting suspected symptoms of TB or SMP. Individuals aged 15 and older had symptom screening, TST, and chest radiography conducted concurrently. TST were performed by staff in accordance with standard guidelines. A qualified nurse administered 0.1 mL (5 IUs) of Purified Protein Derivative (PPD) derived from BCG (Chengdu Institute of Biological Products, Chengdu, China) via intradermal injection into the medial aspect of the left forearm. The size of the induration was assessed 72 h post-injection.

For individuals with suspected symptoms of TB, SMP, or abnormal chest radiography, three sputum specimens were examined by microscopy and cultured. Strain identification and drug susceptibility testing were performed on the cultured positive strains. Suspected cases of TB with negative pathogen results can be further evaluated using computed tomography (CT) and fiberoptic bronchoscopy (FOB). During the outbreak investigation period, given the potential measurement errors in TST testing, Interferon-Gamma Release Assay (IGRA) assays were performed on contacts with moderately Mantoux positive results to more accurately identify those meeting preventive therapy indications. QuantiFERON-TB Gold (Qiagen, Germany) was employed for IGRA testing per manufacturer’s instructions (24). Briefly, whole blood samples were collected in heparinized tubes, incubated with TB-specific antigens (ESAT-6, CFP-10, TB7.7), and plasma interferon-*γ* concentrations were measured by ELISA.

### 2.4 Laboratory testing and MIRU-VNTR typing

According to laboratory test procedures for TB (25), sputum smears were analyzed using the Ziehl-Neelsen staining technique; Mycobacterium TB was cultivated through L-J solid culture and/or BACTEC-MGIT960 liquid culture; drug susceptibility testing was detected by the Roche proportion method, and strains were identified via the traditional biochemical method. Concurrently, GeneXpert was employed for the swift molecular detection of sputum specimens.

We performed mycobacterial interspersed repetitive variable numbers of tandem repeats analyses (MIRU-VNTR) on Mycobacterium TB isolates to determine genetic relationships among isolates, with 9 loci (QUB-11b, QUB-18, QUB-26, MIRU-26, MIRU-31, MIRU-40, Mtub21, Mtub4, and VNTR2372) and 3 hypervariable loci (VNTR3820, VNTR3232, and VNTR4120). We did a cluster analysis of the repeat numbers of 9 VNTR loci of different strains. If 2 or more patients were infected with identical 9 locus genotypes of Mycobacterium TB, they were initially identified as clustered strains. Subsequently 3 hypervariable sites were added to further confirm the recent transmission. If the isolated strains possess specific genotypes at 12 loci, they are classified as a single strain (26). H37Rv standard strain was utilized as a quality control strain.

### 2.5 Statistical analysis

Statistical analyses were performed using the R (version 4.5.0) statistical software. The Chi-square test or Fisher’s exact test was employed, as appropriate, to analyze changes in TST positivity rates between the two screenings and differences in TB incidence rates between Class 1 and Class 2. Differences were declared significant for *p*-value < 0.05.

## 3 Results

### 3.1 Basic characteristics

**Figure 2:**
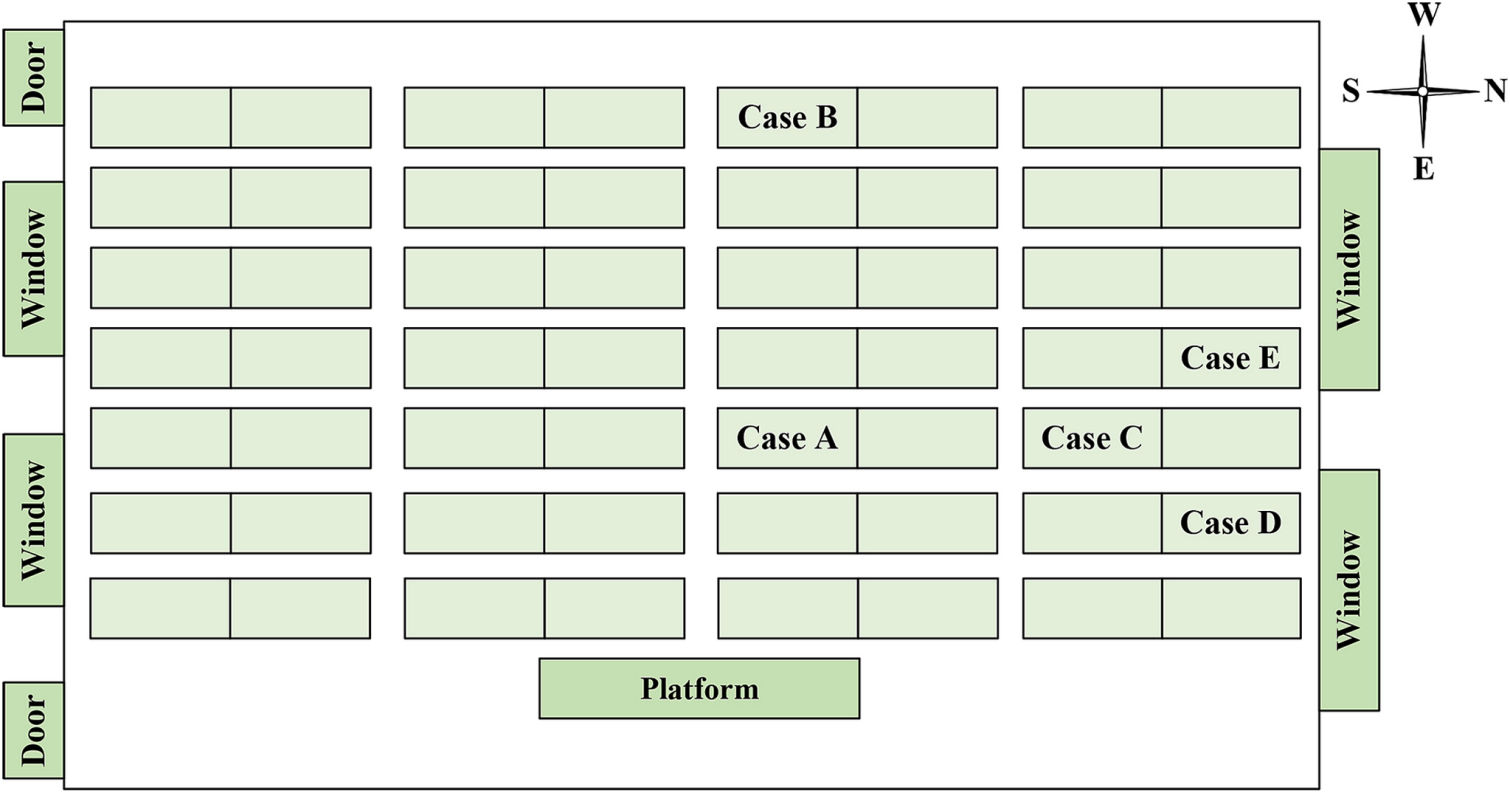
Seat distribution of 5 cases in Class 2.

Between December 2018 and December 2019, a total of six students were diagnosed with TB in two classes of the high school, comprising four boys and two girls. Out of these, five cases were confirmed cases, while one was a clinically diagnosed case (Table 1). The cases included three residential students and three non-residential students, with the dormitories of the residential students being adjacent to each other. Cases B, C, D and E were in the same class (Class 2) as the index case (case A), and the other one (case F) was in the neighboring class (Class 1). The seats of 5 cases in the Class 2 were concentrated on one side of the classroom, and cases C and E were the class cadres (Figure 2). Except for case A, who received home isolation treatment after the diagnosis of TB, cases B-F were isolated at home and received further treatment after being diagnosed as suspected cases.

**Table 1:** Basic information of six cases with TB.

Case	Sex	Age (years)	Class	Residential student (Dormitory)	Symptoms	Recent TST/IGRA	Radiographic findings	Examination of sputum	Date of diagnosis
Case A	F	16	2	N	Cough, expectoration, chest pain	IGRA +	Patchy opacity in the right lower lobe, no cavities detected.	Sputum smear (−), Xpert (−), culture (+)	December 11, 2018
Case B	M	17	2	N	Non	TST:16 mm	Patchy spots, shadows and linear streaky shadows in the right upper lung field, no cavities detected.	Sputum smear (−), Xpert (−), culture (−)	December 30, 2018
Case C	M	17	2	Y (B205)	Non	TST:15.5 mm	Patchy spots, linear streaky shadows and scattered calcified foci in bilateral upper lung fields, no cavities detected.	Sputum smear (−), Xpert (−), culture (+)	January 2, 2019
Case D	M	17	2	Y (B206)	fever, Cough	IGRA +	Patchy high - density shadow in the upper left lung, no cavities detected.	Sputum smear (−), Xpert (−), culture (+)	October 18, 2019
Case E	M	16	2	Y (B207)	Non	TST:9 mm	Patchy and linear blurred shadows in right upper lung field and left lower lobe dorsal segment, no cavities detected.	Sputum smear (−), Xpert (+), culture (+)	October 20, 2019
Case F	F	17	1	N	Non	IGRA +	Patchy opacity in the left lung, no cavities detected.	Sputum smear (−), Xpert (−), culture (+)	November 27, 2019

F, female; M, male; N, no; Y, yes; Xpert, Xpert MTB/RIF; (−), negative; (+), positive.

In September 2019, these students moved from the second year of senior high school to the third. The class number, classmates, dormitory, and roommates remain unchanged; only the classroom location has gone up two floors. The two classes where TB cases were diagnosed have been adjacent all the time, and they were two classrooms located in the horizontal line of the letter “B” (Figure 1). Given that these classrooms were blocked by the classrooms and the offices, we presumed they might have been poorly ventilated.

### 3.2 Diagnosis of the index case

On September 17, 2018, the index case (case A) exhibited clinical symptoms (coughing and expectoration). From October 11th to 27th, due to the worsening symptoms, she went to hospital A for hospitalization and was diagnosed with a suspected case of TB when she was discharged. The hospital reported it on November 11th. Between November 3rd and 9th, Case A was hospitalized at the designated TB hospital B for treatment of a persistent cough and chest pain. Upon discharge, her diagnosis certificate indicated a “lung infection with no active TB present.” On December 11th, the student was diagnosed with TB by hospital B, as the sputum culture tested positive. From the onset of symptoms to the diagnosis of TB, which took nearly three months, Case A continued to attend school regularly, except for the time spent in the hospital. After the diagnosis, the case A was suspended from school and did not return to school until she was cured.

### 3.3 Five screenings for TB among contacts

**Figure 3:**
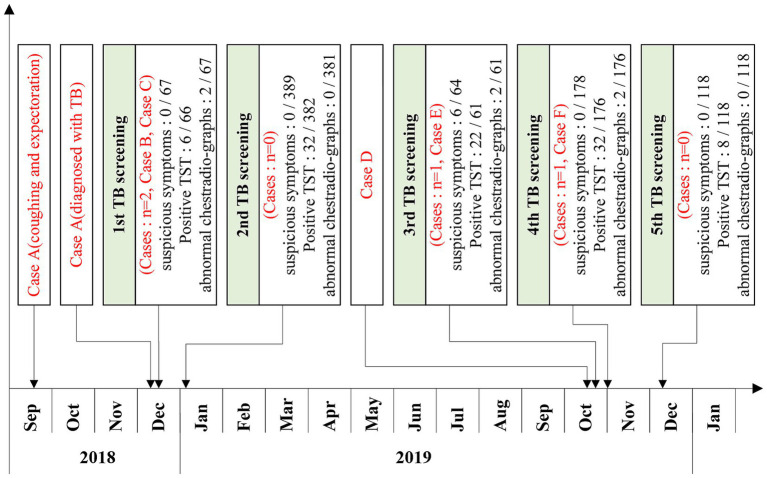
Timeline of the five TB screenings in a high school in Shenzhen.

From December 2018, when the first case was diagnosed, to December 2019, a total of five screenings for TB among contacts were conducted (Table 2; Figure 3).

**Table 2:** Participation and case detection in the five TB screenings.

No.	Screening time	TST screening			Chest radiography screening			Number of detected cases
		Target population	Actual population	Participation rate (%)	Target population	Actual population	Participation rate (%)	
1	December 17, 2018	67	66	98.51	67	67	100.00	2
2	January 4, 2019	389	382	98.20	381	381	100.00	0
3	October 19, 2019	61	61	100.00	61	61	100.00	1
4	November 1, 2019	178	176	98.88	178	176	98.88	1
5	December 9, 2019	118	118	100.00	118	118	100.00	0

In the first round of screening, which commenced on December 17, 2018, 67 close contacts (including 56 classmates and 11 teachers) of Case A were screened. None of them presented with symptoms of TB. TST was performed on 66 close contacts, among whom 3 had SMP. Chest radiography was conducted on 67 close contacts, and two were suspected of having TB. The participation rate of TST screening was 98.51%, and that of chest radiography screening was 100%. Ultimately, this screening identified one clinically diagnosed case (Case B) and one confirmed case (Case C) of TB.

The second round of screening was carried out on January 4, 2019, and the scope of screening was further expanded. Given that case C was a member of the student union, 389 contacts on the same floor and fellow student union members underwent screening. The participation rate of TST screening was 98.20%, and that of chest radiography screening was 100%. No case of TB was found.

On October 1, 2019, a classmate (case D) of case A sought medical care due to symptoms of fever and cough. He was diagnosed as a suspected case of TB by hospital C on October 4 and then transferred to a designated TB hospital (hospital D). He was diagnosed with a confirmed case of TB on October 18.

In the third round of screening, which began on October 19, 2019, 64 close contacts of Case D were screened. Six close contacts exhibited suspected symptoms of TB. TST was performed on 61 close contacts, and none had SMP. Chest radiography was conducted on 61 close contacts, and two were suspected of having TB. The participation rates for both TST screening and chest radiography screening reached 100%. Ultimately, one confirmed case (Case E) of TB was identified in this screening.

On November 1, 2019, the screening scope was broadened to include 178 contacts from the same teaching building and dormitory floor. TST and chest radiography were conducted on 176 contacts. Four contacts had SMP, two had abnormal findings on chest radiography. The participation rates of both TST screening and chest radiography screening were 98.88%. Ultimately, one case (Case F) of TB was diagnosed.

On December 9, the scope of screening was expanded again. 118 contacts in the class near the floor were screened. Both TST screening and chest radiography screening achieved full participation rates of 100%, and no TB was found.

The two classes that found TB cases underwent two rounds of screening in this outbreak. The first screening time of Class 2 was in December 2018 and the second was October 2019. The first screening time of Class 1 was January 2019 and the second was November 2019. The positive rate of TST in the second time of class 2 (35.85, 95% CI: 23.49–19.25%) was higher than that in the first time (8.93, 95% CI: 3.33–20.37%), and the difference was statistically significant (χ^2^ = 11.493, *p* < 0.001). The positive rate of TST in the second time of Class 1 (27.78, 95% CI: 16.86–41.86%) was also higher than that of the first time (14.81, 95% CI: 7.06–27.67%), but the difference was not statistically significant (χ^2^ = 2.707, *p* = 0.100) (Table 3). Five students in Class 2 were diagnosed with TB, yielding an attack rate of 8.77% (95% CI: 2.91–19.30%). Class 1 had 1 confirmed TB case, with an attack rate of 1.85% (95% CI: 0.05–9.89%). The risk of TB in Class 2 was 5.10 times (95% CI: 0.58–45.12) that of Class 1, though this difference did not reach statistical significance (*p* = 0.207). Among the confirmed cases, only 3 students resided in school dormitories, and they were distributed across different dormitories (Dormitory B205, B206, and B207, each housing 8 students), resulting in an attack rate of 12.5% (95% CI: 0.31–52.65%) among dormitory students. No cases were found in other dormitories.

**Table 3:** The positive TST results among screened students.

Screening stage		Class 2				Class 1		
	Screening time	N screened	TST positive students	TST positivity rate	Screening time	N screened	TST positive students	TST positivity rate
First screening	Dec 2018	56	5	8.93% (95% CI: 3.33–20.37%)	Jan 2019	54	8	14.81% (95% CI: 7.06–27.67%)
Second screening	Oct 2019	53	19	35.85% (95% CI: 23.49–19.25%)	Nov 2019	54	15	27.78% (95% CI: 16.86–41.86%)
χ^2^	11.493				2.707			
*P*	<0.001				0.100			

### 3.4 Results of MIRU-VNTR typing

A total of five confirmed cases were reported during this outbreak, among which the sputum-positive culture specimen of one case (Case F) was not obtained. Only 4 cases (case A, C, D, and E) were genotyped by MIRU-VNTR typing. The results showed that all four strains belonged to non-Beijing genotypes. Cases A and C exhibited an identical genotype, and so did Cases D and E. Except for VNTR3232, the copy numbers of 11 loci were identical among Cases A, C, D, and E, indicating their belonging to the same transmission chain (Table 4).

**Table 4:** Results of MIRU-VNTR typing in 4 patient strains.

Case	QUB-11b	QUB-18	QUB-26	MIRU-26	MIRU-31	MIRU-40	Mutb21	Mtub4	VNTR2372	VNTR3820	VNTR3232	VNTR4120
Case A	3	3	7	5	3	3	4	2	2	6	7	5
Case C	3	3	7	5	3	3	4	2	2	6	7	5
Case D	3	3	7	5	3	3	4	2	2	6	9	5
Case E	3	3	7	5	3	3	4	2	2	6	9	5

### 3.5 Preventative treatment and follow-up observation

Following two rounds of screenings conducted between December 2018 and January 2019, five contacts with SMP were found. Regrettably, all declined to undergo preventative medication. From October to December 2019, 29 contacts were moderately positive and 4 were SMP by three screenings. In order to further clarify the infection status, 29 moderately positive contacts were given IGRA, and 10 contacts were positive. Fourteen contacts who were SMP or IGRA positive were strongly recommended and mobilized to take prophylactic medication. Only one student accepted and completed prophylactic medication. For this outbreak, the overall tuberculosis preventive treatment (TPT) acceptance rate was 5.26% (1/19), including an 8.33% (1/12) acceptance rate among LTBI conversion participants. Teachers and students who declined prophylactic medication were mandated to undergo chest radiography at the conclusion of the 3rd, 6th, and 12th months post-screening. Continue to strengthen the follow-up observation of the classes where the outbreak occurred, no new related cases were found until November 2024.

## 4 Discussion

A cluster outbreak of TB in a high school in Shenzhen, Guangdong Province, China, was reported in our study. The potential causes of the outbreak and the possible source of transmission were investigated based on the results of the epidemiological investigation, clinical data, and laboratory testing.

In this outbreak, despite an approximate 1-year interval between the identification of cases A, B, and C and that of cases D, E, and F, epidemiological investigation and MIRU-VNTR typing results indicated that all TB patients belonged to the same transmission chain. Based on the investigation results, we put forward several insights.

Our investigation revealed that delays in reporting and diagnosing of the index case may have been the primary factors contributing to the spread of TB among students, which is consistent with previous studies (9, 27). In accordance with the TB prevention and control policies for schools in China, students suspected of having TB were required to cease attending classes until TB was ruled out (23). However, when the index case (case A) was initially diagnosed as suspected TB, the doctor failed to report it in a timely manner. Pediatric TB is characterized by atypical clinical symptoms and imaging changes, insidious onset, and a low positive rate of etiological detection, all of which increase the difficulty of diagnosing childhood TB (28–30). Moreover, due to inadequate physician awareness of pediatric TB and the limitations of laboratory diagnostic methods, case A was diagnosed with “pulmonary infection” after hospitalized in the TB-designated hospital. Consultation and diagnostic delay for her lasted for nearly 3 months, during which she attended and lived at the school, continuing close contact with her classmates. Delayed case reporting and diagnostic inaccuracies resulted in prolonged exposure of infectious individuals within congregate settings, facilitating *Mycobacterium tuberculosis* transmission and triggering additional student cases. This outbreak not only compromised students’ physical and mental health but also disrupted their academic progression. This further suggests that designated medical institutions for TB should be cautious in diagnosing TB. For patients with TB who temporarily lack etiological results or have negative etiological tests, especially those in special places such as students, it is recommended to establish a joint diagnostic team. The joint diagnostic team would be responsible for the diagnosis and periodically discuss cases to promptly correct misdiagnoses (31, 32), in order to maximize the accuracy of the diagnosis as much as possible.

Inadequate classroom ventilation (inferred from building design and seasonal conditions) and insufficient implementation of routine TB prevention and control measures in schools may have contributed to the increased TB transmission (14, 33). The teaching building where the current outbreak took place was in the shape of the letter “B,” with the two affected classrooms situated on the horizontal line of the “B.” Owing to the influence of the surrounding classrooms and offices, the ventilation was poor. Furthermore, the outbreak occurred in winter, and classroom doors and windows remained closed throughout lessons, which was not conducive to air circulation. The classroom was equipped with ultraviolet disinfection lamps. However, the installation height of the lamps was excessively high, and their surfaces were covered with a significant amount of dust, so it was difficult to guarantee the effect of air disinfection.

Our study further disclosed that the screening period for some close contacts with a negative TST might concur with the window period of latent tuberculosis infection (LTBI) (34). Concentrating medical observation and intervention exclusively on close contacts exhibiting SMP could potentially miss some covert secondary cases, thus undermining the efficacy of epidemic prevention and control endeavors. In previous studies, secondary cases often had SMP in the first (or previous) TST screening (10, 35). In contrast, in this outbreak, the subsequent confirmed cases (Cases D, E, and F) had negative or generally positive TST results in both the first and second screenings. Moreover, none of the teachers and students who had SMP in the first and second screenings developed the disease during subsequent follow-up. China remains not only a high-TB-burden nation but also ranks among the most severely LTBI-affected countries worldwide (36, 37). The detection of SMP among teachers and students may not be indicative of recent infection. On the contrary, teachers and students with recent infections may be in the window period of LTBI, resulting in negative TST results. The significant increase in the TST positivity rate in the affected class 10 months later further confirms the existence of the LTBI window period. Therefore, in the management of TB outbreaks within schools or collective institutions, the establishment of long-term monitoring and secondary screening mechanisms for close contacts should be accorded high priority. Particular vigilance should be exercised toward individuals who were not found to be infected with LTBI during the initial screening phase. Regrettably, according to China’s School Tuberculosis Prevention and Control Work Regulations (2017 Edition) (23) in effect at that time, mandatory secondary screening 2–3 months later was only mandated in public health emergencies. However, follow - up screening was not performed in this case, which failed to promptly identify individuals newly infected with TB or with LTBI.

Notably, our study also found a low acceptance rate (5.26%) for TPT among teachers and students. This rate was lower than the acceptance rate (10.81%, 4/37) observed among an outbreak of pulmonary tuberculosis in a high school in an eastern city in China, 2016–2019 by Fang et al. (38) and the rate (16.99%, 183/1077) reported in a multicenter study in China by Ren et al. (39), representing a substantial gap from the 90% target set by the United Nations High-Level Meeting on TB (40). Upon investigation, the main reasons for the refusal of TPT by teachers and students were identified as the low level of awareness regarding the hazards of the disease and the necessity of TPT. Additionally, as students were approaching the college entrance examination, parents were apprehensive about potential adverse reactions caused by medication. These factors have also been identified in previous studies (41–43). This suggests that there is a need to adopt personalized health education approaches to enhance the acceptance of TPT among the population.

This study has a few limitations. Firstly, due to the failure to obtain the sputum positive culture of case F, the recent transmission relationship between this case and other positive cases cannot be confirmed by genotyping. However, the outbreak occurred in a relatively closed campus environment, and the results of on-the-spot epidemiological investigation can reflect the spread of the outbreak to a certain extent. In addition, although MIRU-VNTR typing with 12 loci confirmed recent transmission among cases, its moderate discriminatory power may underestimate strain diversity in larger outbreaks. Future studies should prioritize whole-genome sequencing for higher resolution. Secondly, environmental assessments were qualitative; future outbreak investigations should incorporate quantitative ventilation metrics to objectively evaluate transmission risks. Thirdly, given China’s near-universal BCG vaccination coverage, individual vaccination status wasn’t recorded. Taking into account simultaneously the small sample size of confirmed cases (n = 6) and contacts (Class 2: n = 56; Class 1: n = 54), along with limited age variation (<1 year) due to school enrollment policy, we omitted adjustment for potential confounders such as age or BCG vaccination status, potentially affecting precision. Future studies should consider multivariable regression or stratified analyses to better assess causal relationships. Fourthly, contact follow-up relied on China’s Tuberculosis Information Management System, which achieves nationwide TB case reporting coverage but remains subject to underreporting errors that may bias transmission risk estimates. Post-graduation geographic dispersion precluded systematic follow-up. Future studies should explore implementing telephone-based surveillance with additional personnel to verify contact disease status.

## 5 Conclusion

In conclusion, the delayed reporting and misdiagnosis of the index cases, inadequate classroom ventilation, and insufficient implementation of routine prevention and control measures at the school were contributing factors to this outbreak. The TB-designated hospitals should exercise caution in diagnosing TB, especially among special populations such as students, and report cases promptly as required. In the management of TB outbreaks in schools, it is imperative to establish long-term monitoring and secondary screening mechanisms for close contacts to rule out recent infections that may have been missed due to the window period. Routine prevention and control measures at schools should be implemented, and health education for teachers and students should be strengthened.

## Data Availability

The original contributions presented in the study are included in the article/supplementary material, further inquiries can be directed to the corresponding author/s.
